# Antiretroviral Drug-Associated Oral Lichenoid Reaction in HIV Patient: A Case Report

**DOI:** 10.1155/2010/291072

**Published:** 2010-07-05

**Authors:** Pratanporn Arirachakaran, Mattana Hanvanich, Piyanad Kuysakorn, Kobkan Thongprasom

**Affiliations:** ^1^Infectious Diseases Clinic, Chulalongkorn University Dental Hospital, Chulalongkorn University, Bangkok 10330, Thailand; ^2^Department of Oral Medicine, Faculty of Dentistry, Chulalongkorn University, 34 Henri Dunant Road, Pathumwan District, Bangkok 10330, Thailand; ^3^Division of Infectious Diseases, Department of Medicine, King Chulalongkorn Memorial Hospital, Faculty of Medicine, Chulalongkorn University, Bangkok 10330, Thailand

## Abstract

Antiretroviral therapy has changed the course of HIV disease and improved quality of life in HIV patients. Incidence of an oral lichenoid drug reaction induced by zidovudine is not common. Once it occurs, it affects a patient's well being, in particular their oral functions. Here we report the first case of a 34-year-old Thai man with painful erosive lesions involving the lip and buccal mucosa. Treatment with topical fluocinolone acetonide 0.1% alleviated the patient's oral pain, but it was not until the subsequent withdrawal of zidovudine that the patient showed improvement and resolution of the lesions. Long-term follow-up was useful in the management of this patient, and no recurrence of the lesion was found during 21-month follow-up in this patient.

## 1. Introduction

Oral lichenoid drug reaction (OLDR) is not an infrequent occurrence following initiation of certain drug regimens such as antihypertensive and antiretroviral medications. OLDR lesions clinically present as white striae, papules, plaques with erythema, or erosion of the oral mucosa. The severity of symptoms can vary from burning sensation to severe pain interfering with a patient's oral functions. OLDR is difficult to distinguish clinically, with identical presentation in biopsies, from oral lichen planus (OLP). A thorough medical history, especially medications taken and distinct clinical appearance with subsequent resolution following drug cessation are essential in an accurate diagnosis of OLDR [1].

HIV-infected patients who commence on antiretrovirals need life-long treatment. The antiretrovirals (ARVs) reduce viral load and increase CD4^+^ T cell count thereby slowing disease progression and improving the patients' quality of life [2]. Since the approval of AZT by the US FDA in 1987, it has been widely used for the treatment of human immunodeficiency virus infection. In Thailand, highly active antiretroviral therapy (HAART) is usually composed of zidovudine (AZT) or stavudine (d4T) + lamivudine (3TC) plus nevirapine (NVP) or efavirenz (EFV) [3]. During the course of ARVs administration, the patients may experience adverse drug effects [4–6]. The orofacial adverse effects of HAART including oral ulcers, xerostomia, mucositis, hyperpigmentation, erythema multiforme (EM), cheilitis, perioral paresthesia, angioedema, and taste alteration have been reported [7]. To our knowledge, there has been only one report of AZT-induced lichenoid reaction [8]. In this report, we describe the first Thai HIV patient presenting with oral lichenoid drug reaction after receiving HAART containing AZT which resolved following drug removal.

## 2. Case Report

A 34-year-old man was referred to the Faculty of Dentistry, Chulalongkorn University, Bangkok, Thailand from the Department of Medicine, King Chulalongkorn Memorial Hospital with a chief complaint of a painful and burning sensation of lip and oral mucosa when consuming food. The patient was an ex-intravenous drug user who had acquired HIV and hepatitis C (HCV) genotype 1 infections for 12 years possibly from multiple heterosexual partners or intravenous drug use. He had tuberculosis (TB) of lymph gland and had been treated with anti-TB drugs for one year. Six months after the TB treatment, HAART was started with daily doses of 600 mg AZT, 300 mg 3TC, and 800 mg of EFV. Monitoring of drug efficacy was assessed periodically. Baseline plasma HIV-1 RNA viral load (VL) was 97,500 copies per mL (4.99 log) before commencing HAART. He responded quite well to the treatment and the HIV-VL fell below the limit of detection of less than 50 copies per mL after 6 months of treatment. The patient's immune status was improved with the absolute CD4^+^ T cell count at baseline, 6 months, and 30 months as 31, 168, and 287 cells per *μ*L, respectively. 

**Figure 1:**
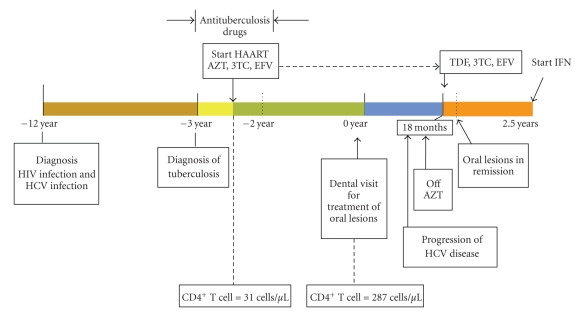
The time line of drug administration, occurrence of the oral lesions, withdrawal of the AZT, and remission of the lesions.

**Figure 2:**
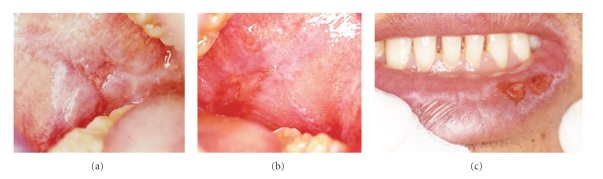
The oral lesions at the first visit: (a) right buccal mucosa, score 5, (b) left buccal mucosa, score 5, and (c) lip, score 4. Note the white striae and papules with deep ulceration on the erythematous base.

**Figure 3:**
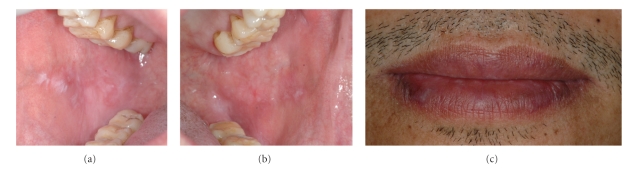
Oral lesions improved at 21 months: (a) right buccal mucosa, score 1, (b) left buccal mucosa, score 0, and (c) the lip, score 0.

The patient denied drug hypersensitivity, smoking, and alcohol consuming habits. The oral lesions developed two and a half years after beginning HAART. Oral examination revealed erosive lesions of the lip and right and left sides of buccal mucosa. The lesions had slightly raised keratotic white reticular striae and white papules on an extensive background of erythematous mucosa with deep ulcerations (Figures 2(a), 2(b), and 2(c)). Clinical scores of the right and left buccal mucosa as well as the lip lesions were 5, 5, and 4, respectively [9]. Both lateral sides of the tongue displayed nonpainful and very mild white plaques. Areas of redness corresponding to the sharp edges of the adjacent teeth were also observed. The OLDR diagnosis of lesions at the lip and buccal mucosa was based on the history of medications and the incidence of initial eruptions of the lesions as shown in Figure 1. Management in this patient was focused on relieving the symptoms. Topical fluocinolone acetonide 0.1% orabase, solution, and sodium bicarbonate mouthwash were prescribed. Smoothing of sharp edges area of teeth resolved in healing of the red area at lateral sides of the tongue, and long-term follow-up was planned. While the use of topical steroid relieved the patient of oral pain, the lesions themselves did not resolve. After treating the oral lesions for 19 months, a liver biopsy was performed and showed fibrosis stage 3 from chronic HCV type 1 hepatitis. Pegylated interferon and ribavirin were planned to be prescribed to the patient in management of the HCV infection. Due to the myelosuppressive effect of AZT, it was decided to withdraw the patient from AZT prior to interferon administration. AZT was switched to tenofovir (TDF) 300 mg, but EFV and 3TC were continued. Two months after the withdrawal of AZT and with the local topical steroid treatment, only very mild white papules at right buccal mucosa remained, and the clinical pain score at this site was 1, while other sites were in complete remission (Figure 3). Pegylated interferon and ribavirin therapy began 1 year after the cessation of AZT.

## 3. Discussion

Here we report the first case of a 34-year-old Thai HIV positive man receiving HAART (AZT, 3TC, and EFV), presenting with painful erosive lesions involving the lip and buccal mucosa. While topical steroid application lessened his pain, it was only upon removal of AZT from his drug regimen with all other factors constant that his lesions improved, indicating a diagnosis of OLDR.

OLDR induced by AZT is possibly caused by the CD8^+^ T cells previously primed from previous antigens. CD8^+^ T cells become trapped and persist at the mucosal site, and later become effectors of mucosal damage after cross-reacting with the drug [10].

Histopathological study is one method used to confirm the OLDR diagnosis, but it was not possible in this case since the patient refused a biopsy. However, the diagnosis was based on a thorough history of medications taken, and a typical oral presentation, with lesions resolving subsequent to cessation of the drug. AZT-induced OLDR was previously reported in 8 cases since the introduction of this drug in treating HIV patients by Ficarra [8]. Xerostomia and EM can be found in patients taking 3TC and EFV [7], while mucocutaneous hyperpigmentation and EM are common orofacial effects of AZT [7]. EM was easily excluded because EM usually has an acute onset, while in this patient the onset was over 2 years after HAART began. Furthermore, obvious Wickham's striae were observed in the lesions. Although hairy leukoplakia (HL) was detected at the lateral sides of the tongue in this patient, the lesions at the lip and buccal mucosa could be easily discriminated from HL by the clinical characteristics of reticular raised white striae. 

The relationship between OLP and hepatitis C virus (HCV) infection has been reported in Thai patients with OLP [11]. A thorough medical history and a long-term follow-up showed the oral lesions remitted around the time the patient was withdrawn from AZT. The treatment of HCV infection with pegylated interferon and ribavirin began 9 months after the remission of oral lesions; so it is quite unlikely that these played a role in this case. Candida infection and smoking related leukoplakia were also in the differential diagnosis but were ruled out. Hyperkeratotic lesions caused by human papilloma virus (HPV) were also excluded because HPV infection normally displays as either multiple sessile or dome-shape lesions depending on the type of HPV, but without ulceration. Furthermore, there was a remission of the lesions after discontinuing the drug. 

Various drugs have been reported to cause lichenoid reaction, including isoniazid, one of the antituberculosis drugs [12], but the OLDR did not develop until 2 years after the withdrawal of isoniazid in this patient. Ficarra reported that OLDR developed 8–24 weeks after the initiation of AZT or ketoconazole [8]. In this case, OLDR lesions appeared 2.5 years after initiation of the HAART. OLDR was seen to develop after administration of drugs varied from 1 month to a year in one study [13]. In our case, remission resulted two months after the withdrawal of AZT. In OLDR it is common to observe improvement of the lesions at least a month after withdrawal of the suspected drug [14, 15]. To determine with absolute certainty that AZT was the cause of this patient's lesions would require rechallenge, or provocation with the drug. The ethics of such a course of action are extremely questionable, given the severity of this patient's lesions. Treatment with topical fluocinolone acetonide 0.1% alleviated the patient's oral pain, but it was not until the subsequent withdrawal of zidovudine that the patient showed improvement and resolution of the lesions.
